# Surrogate endpoint for overall survival in assessment of adjuvant therapies after curative treatment for hepatocellular carcinoma: a re-analysis of meta-analyses of individual patients’ data

**DOI:** 10.18632/oncotarget.18853

**Published:** 2017-06-29

**Authors:** Hong-Bo Huan, Li-Li Wu, Wan-Yee Lau, Xu-Dong Wen, Liang Zhang, Da-Peng Yang, Xi-Shu Wang, Ping Bie, Feng Xia

**Affiliations:** ^1^ Institute of Hepatobiliary Surgery, Southwest Hospital, Third Military Medical University, Chongqing, China; ^2^ Faculty of Medicine, The Chinese University of Hong Kong, Prince of Wales Hospital, Shatin, Hong Kong SAR, China

**Keywords:** hepatocellular carcinoma, overall survival, disease-free survival, surrogate endpoint

## Abstract

The gold standard endpoint to evaluate the effect of treatment for hepatocellular carcinoma (HCC) is overall survival (OS), but it requires a longer follow-up period to observe. This study aimed to identify whether disease-free survival (DFS) could be used as a surrogate endpoint for OS to assess the efficacy of adjuvant therapies after curative treatment (surgical resection and ablation) for HCC patients. A systematic review was conducted to identify trials about curative treatment combined with or without adjuvant therapies (interferon, IFN; or transarterial chemoembolization, TACE) for HCC. Total of 2211 patients’ data from 17 trials were analyzed. At the individual study level, DFS was strongly correlated to OS (*ρ* = 0.988 and 0.930, 95% CI: 0.965–0.996 and 0.806–0.976 for the studies comparing Radiofrequency ablation (RFA) + TACE to RFA alone; and for the studies comparing curative treatment + IFN to curative treatment alone, respectively). At the trial level, the effects of treatment on DFS and OS were also strongly correlated to each other (R = 0.815 and 0.854, 95% CI: 0.536–0.934 and 0.621–0.948, respectively). In conclusion, DFS could be used as a potential surrogate endpoint for OS to assess the effect of adjuvant therapies after curative treatment for HCC.

## INTRODUCTION

Liver cancer is the second most common cause of cancer-related death, with 782,500 new cases and 745,500 cancer deaths occurred in 2012 worldwide [1]. As the major histological subtype, hepatocellular carcinoma (HCC) occupies 70% to 85% of patients with liver cancers [2]. During the past decades and with advances in diagnosis and treatment, the median survival reaches beyond 5 years [3]. With further exploration of treatment for HCC, overall survival (OS) as a standard endpoint to evaluate the efficacy of treatment is complicated, because it requires a large number of patients and longer follow-up period. It is necessary and meaningful to explore a reliable surrogate endpoint for OS to allow early assessment of treatment for HCC

Treatment of HCC has changed greatly within the past decades and become a major research area [3]. For the treatment of HCC, surgical resection, liver transplantation and local ablative therapy are considered to be the curative treatment [4]. Surgical resection is preferred choice for HCC without cirrhosis [3]. HCC patients with normal concentration of bilirubin and no portal hypertension, the probability that survival time reaches 5 years is 70% after surgical resection [5]. Radiofrequency ablation (RFA) is commonly recommended as a first line local ablative therapy for tumors less than 5cm [3]. After RFA, survival in Child-Pugh A patients was 50–75% at 5 years [6, 7]. In addition, transarterial chemoembolization (TACE) and interferon (IFN) as the non-curative treatments have been confirmed to improve survival [8, 9]. TACE induces objective responses in 35% patients and improves 2-year survival [8, 10]. It has been confirmed that IFN decreased the rates of tumor recurrence and mortality for hepatitis B virus (HBV) or/and hepatitis C virus (HCV) related HCC [9, 11]. Pre- and post-operative antiviral and anti-inflammatory treatment with IFN has been shown to reduce early and late tumor recurrence rates in HCC patients with HBV or/and HCV infection [11, 12]. In order to find out more effective treatment to improve survival, the combined utilization of adjuvant therapy after curative treatment has attracted increasing attention. Meanwhile, it requires strict evaluation criteria to study the effect of adjuvant therapies.

Overall survival as the gold standard endpoint being used to assess the effect of HCC treatment is reliable. However, it requires a large number of patients and longer follow-up period to estimate significant differences between groups of patients [13]. Utilization of a surrogate endpoint at an early stage in clinical trials could speed up assessment of efficacy and reduce costs. It was defined that OS is the time from randomization to death from any cause, and disease-free survival (DFS) was the time from randomization to the first event (loco-regional, distant recurrence, or death from any cause) after treatment [14]. Recent studies had confirmed that DFS is a valid surrogate endpoint for OS in the clinical trials for the treatment of colon cancer, gastric cancer, and lung cancer [14–16]. However, there is still no available surrogate endpoint for OS to assess the efficacy of adjuvant therapies in HCC study. The purpose of our study was to evaluate whether DFS could be used as an early surrogate endpoint in studies involving adjuvant therapies after curative treatment for HCC patients.

## RESULTS

This study is based on the individual study data of 2211 patients in 17 studies that were included in 8 meta-analyses. The main characteristics about 7 meta-analyses for RFA + TACE *vs.* RFA and 1 meta-analysis for curative treatment + IFN *vs.* curative treatment were summarized (Table 1). After reviewing 116 trials including 10069 patients from 7 meta-analyses, repeated trials and patients, as well as the studies failed to get HRs for OS and DFS were excluded, 7 trials containing 1042 patients were conformed to the inclusion criteria for RFA + TACE *vs.* RFA (Table 2). The main characteristics were summarized in Table 2. For curative treatment + IFN *vs.* curative treatment, 10 trials containing 1169 patients were conformed to the inclusion criteria after elimination of unqualified and duplicate data (Table 3). The main characteristics were summarized in Table 3. The HRs for OS and DFS were either obtained directly or through the Kaplan-Meier survival curves in these studies.

**Table 1:** The eligible eight meta-analyses included in this study

Year	Author	Content of study	Type of studies included	No. of studies	No. of patients	Type of data
2014	Gu L [17]	TACE + RFA/PEI/HIFU/PAI *vs.* TACE/RFA	RCT, CS	18	2120	RR
2014	Jiang G [18]	RFA + TACE *vs.* RFA	RCT, CS	19	1728	OR
2014	Kong QF [19]	RFA + TACE *v*s. RFA	RCT, CS	19	1728	OR
2013	Lu Z [20]	RFA + TACE *v*s. RFA	RCT	7	574	OR
2013	Liao M [21]	TACE+RFA/PEI/RT/3D-CRT/HIFU *vs.* TACE	RCT, PS, RS	28	1815	RR
2013	Ni JY [22]	RFA + TACE *vs.* RFA/TACE	RCT	6	376	OR
2012	Yan S [23]	RFA + TACE *vs.* RFA	RCT, CS	19	1728	OR
2014	Zhang W [24]	CT + IFN *vs.* CT	RCT, CCS	14	1835	RR

RCT: randomized controlled trial; PS: prospective study; RS: retrospective study; CS: cohort study; CCS: case-control study; OR: odds ratio; RR: risk ratio; RFA: radiofrequency ablation; TACE: transcatheter arterial chemoembolization; PEI: percutaneous ethanol injection; PAI: percutaneous acetic acid; HIFU: high-intensity focused ultrasound; 3D-CRT: three-dimensional conformal radiation therapy; CT: curative treatment; IFN: interferon.

**Table 2:** The trials for RFA + TACE vs. RFA

Year	Author	Type of study	No. of patients			Follow-up (months)
			RFA + TACE	Total		
2013	Peng ZW[25]	RCT	94	95	189	7–62
2012	Kim JW[26]	RS	83	231	314	0–108
2012	Peng ZW[27]	RCT	69	70	139	0–103
2010	Morimoto M [28]	RCT	19	18	37	12–46
2010	Peng ZW [29]	CCS	120	120	240	0–64
2009	Shibata T [30]	RCT	46	43	89	9–68
2005	Shen SQ [31]	CCS	18	16	34	5–38

RCT: randomized controlled trial; CCS: case-control study; RS: retrospective study; RFA: radiofrequency ablation; TACE: transcatheter arterial chemoembolization.

**Table 3:** The trials for CT + IFN vs. CT

Year	Author	Type of study	No. of patients			Follow-up (months)
			CT+IFN	CT	Total	
2012	Chen LT [32]	RCT	133	135	268	0–66.9
2011	Hagihara H [33]	CCT	37	145	182	0–120
2007	Lo C M [34]	RCT	40	40	80	0–160
2007	Jeong SC [35]	CCT	42	42	84	0–144
2007	Jeong SC [36]	CCT	16	16	32	0–45
2007	Kudo M [37]	CCT	43	84	127	0–100
2006	Sun HC [38]	RCT	118	118	236	0–68
2003	Shiratori Y [39]	RCT	49	25	74	0–108
2002	Miyaguchi S [40]	CCT	22	24	46	0–45
2001	Suou T [41]	CCT	18	22	40	0–110

RCT: randomized controlled trial; CCT: case-cohort study; CT: curative treatment; IFN: interferon.

**Figure 1:**
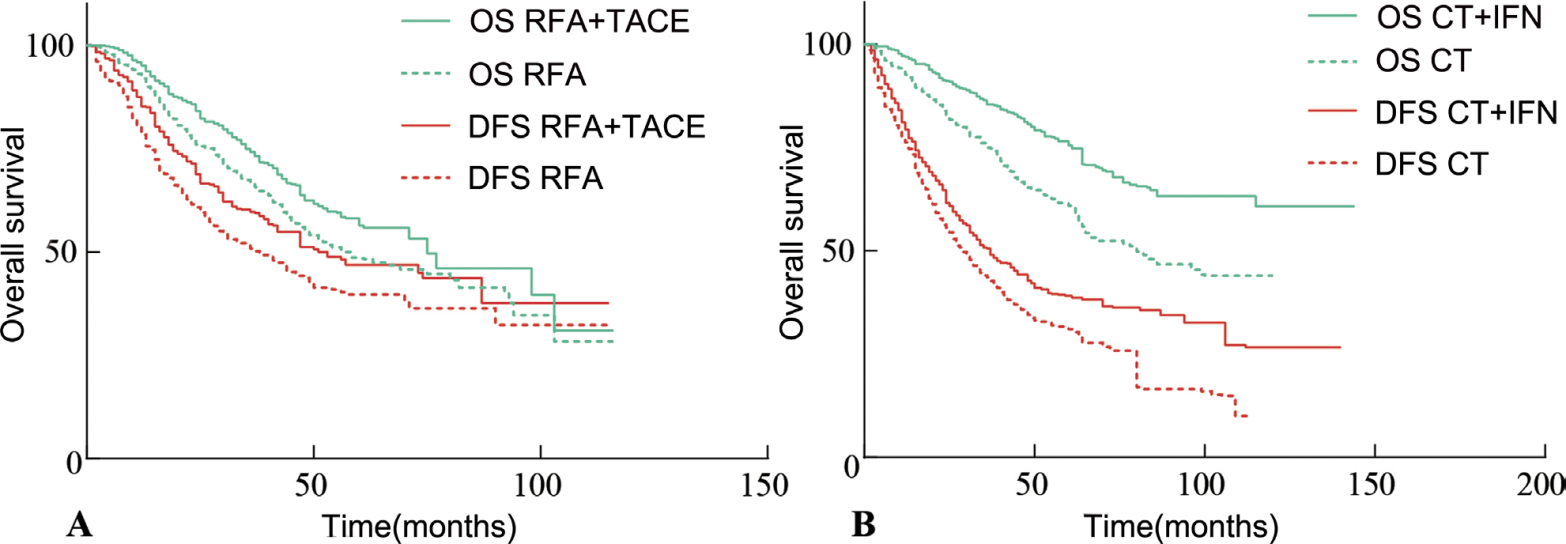
Kaplan-Meier curves of DFS and OS in assessment the effect of adjuvant therapies after curative treatment for HCC patients (**A**) Assessment of RFA + TACE *vs.* RFA. (**B**) Assessment of curative treatment + IFN *vs.* curative treatment. OS = overall survival. RFA = Radiofrequency ablation. TACE = Transarterial chemoembolization. DFS = disease-free survival. CT = curative treatment.

For treatment with RFA + TACE *vs.* RFA trials, a total of 7 trials containing 1042 patients were available for analysis. High correlation between the treatment effects on DFS and OS was observed, with a rank-correlation coefficient ρ equaled to 0.988 (95% CI, 0.965–0.996; Figure 1A). The 10 curative treatment-based trials (curative treatment and combination IFN) containing 1169 patients exhibited a high correlation between treatment effects on DFS and OS, with a rank-correlation coefficient ρ equaled to 0.930 (95% CI, 0.806–0.976; Figure 1B).

**Figure 2:**
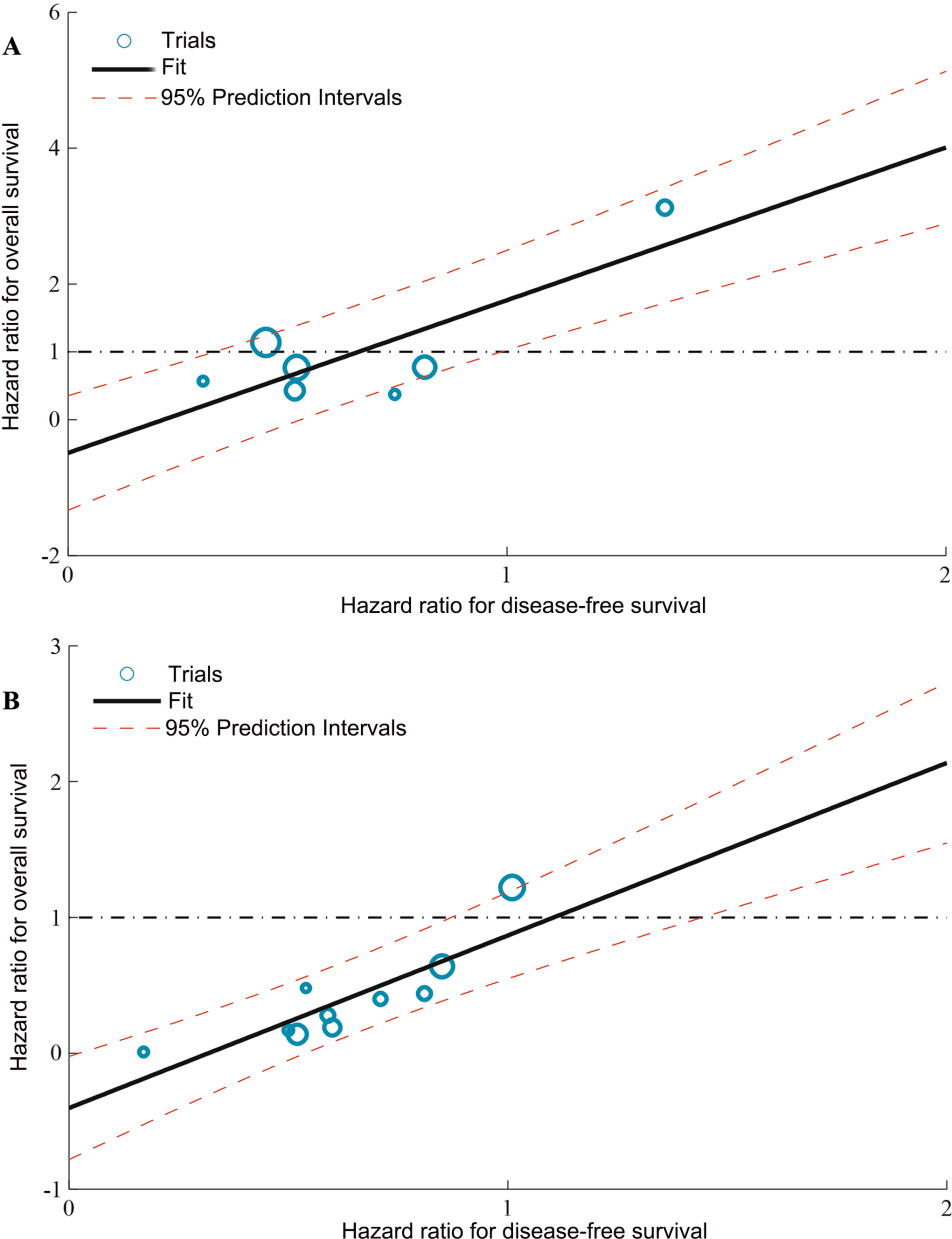
Correlation between treatment effects on DFS and OS (**A**) Assessment of RFA + TACE *vs.* RFA. (**B**) Assessment of curative treatment + IFN *vs.* curative treatment. Each trial is represented by a circle with a size proportional to the number of patients. The black solid line corresponds to the fitted regression line and the red dashed lines correspond to 95% CI. Correlation values are good (R = 0.815 and 0.854). CT = curative treatment.

According to the individual study data from 17 trials, the HRs on the endpoints was appraised. Linear regression analysis was carried out to analyze the correlation between treatment effects on DFS and OS in RFA + TACE *vs.* RFA trials and curative treatment + IFN *vs.* curative treatment trials, respectively, and it revealed strong correlation between DFS and OS (Figure 2A, 2B). The correlation coefficient R between the HRs were 0.815 (95% CI 0.536–0.934) for RFA + TACE *vs.* RFA (Figure 2A), and 0.854 (95% CI 0.621–0.948) for curative treatment + IFN *vs.* curative treatment (Figure 2B).

Based on the linear model, as the minimum treatment effect on the surrogate endpoint (DFS), the surrogate threshold effects (STE) is necessary to calculate for predicting a non-zero effect on OS. The STE (based on the estimation error adjusted prediction limits) for RFA + TACE *vs.* RFA alone corresponded to a DFS HR of 0.33 (for a beneficial treatment) or 0.99 (for a harmful treatment; Figure 2A). In addition, for curative treatment + IFN *vs.* curative treatment alone, the STE corresponded to a DFS HR of 0.87 (for a beneficial treatment) or 1.44 (for a harmful treatment; Figure 2B).

**Figure 3:**
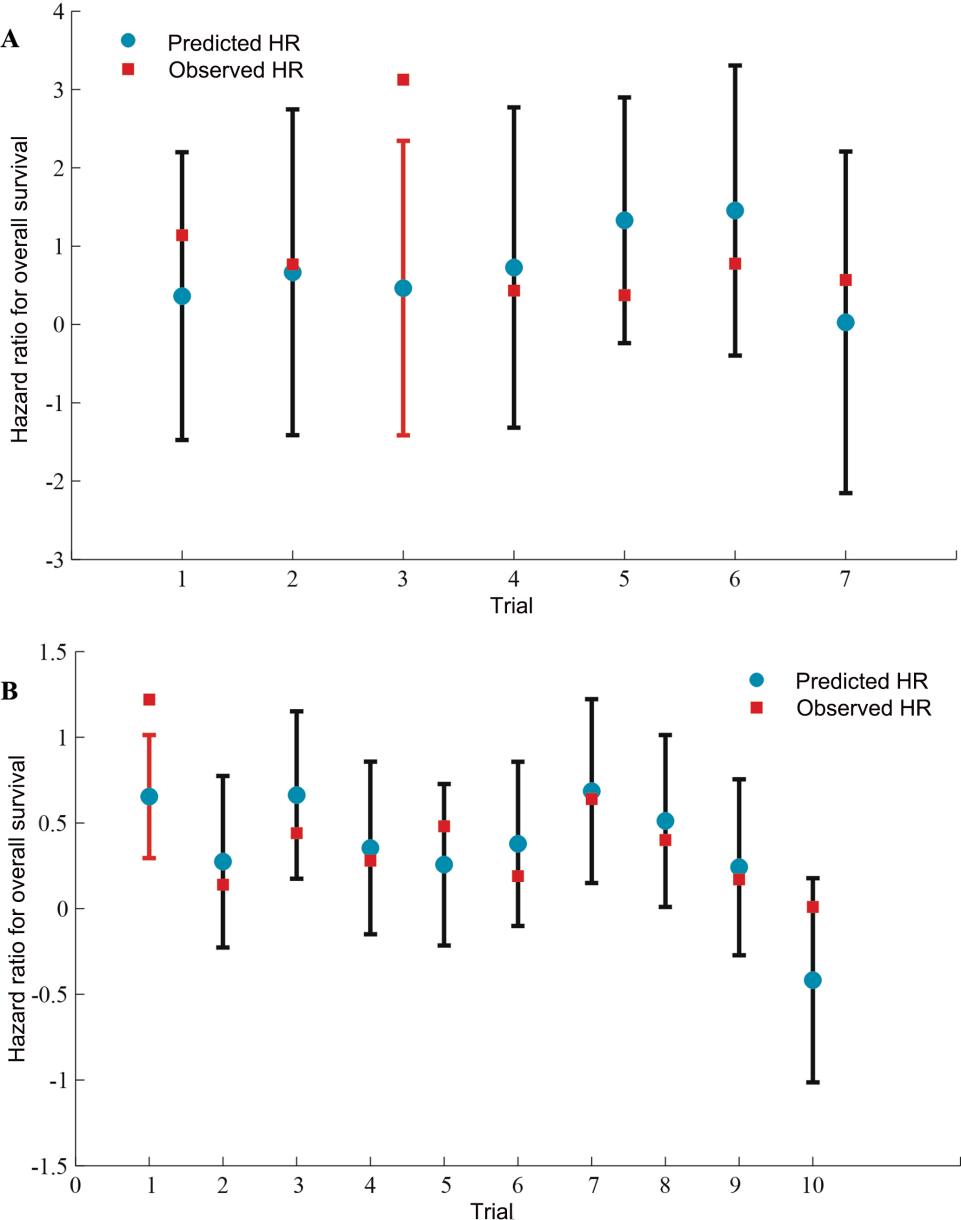
Internal validation of the prediction of OS by treatment effects on surrogate endpoints (**A**) Assessment of RFA + TACE *vs.* RFA. (**B**) Assessment of curative treatment + IFN *vs.* curative treatment. The blue circles correspond to the predicted hazard ratios for overall survival using the observed hazard ratio on disease-free survival of that particular trial and the surrogate model built on all the other trials; vertical lines correspond to 95% prediction intervals; the red squares correspond to observed hazard ratios on overall survival; Predicted values from trials for which observed hazard ratios are outside the limits are in red. HR = hazard ratio. CT = curative treatment.

The prediction results from the leave-one-out cross-validation analysis showed that for DFS, the observed HRs for OS fell between the limits of the 95% prediction intervals in 6 of the 7 studies for RFA + TACE *vs.* RFA alone and 9 of the 10 studies for curative treatment + IFN *vs.* curative treatment alone (Figure 3A, 3B).

## DISCUSSION

Fast progress and improvement in treatment has prolonged survival of patients with HCC. However, the best treatment for patients with different stages of HCC remains to be explored. There is no doubt that the best endpoint to be used in these studies is OS, but it requires a longer follow-up period. It has been reported that the time to progression (TTP), closely related to DFS, could be used as a surrogate endpoint for OS in the clinical trials of advanced HCC [42]. In present study, our results showed that the effects of treatment on DFS and OS were strongly correlated in the HCC patients treated with curative treatment (surgical resection and ablation) combined with or without TACE and IFN. Both the correlation coefficient R (trial-level) and the rank-correlation coefficient ρ (individual-level) are close to 1 between DFS and OS in these patients, thus DFS is a validated surrogate endpoint for OS statistically [43]. These findings suggest that DFS could be used as a surrogate endpoint for OS to reduce the duration and cost in the future clinical studies.

As a reasonable candidate for a surrogate of OS, DFS has been confirmed that it is a surrogate for OS in many tumor types [14–16]. Moreover, it has been reported that DFS was the preferred potential surrogate endpoint for small HCC according to a questionnaires survey among clinicians and methodologists [44]. Our study confirmed that DFS could be used as a surrogate endpoint for OS to accelerate assessment of efficacy for adjuvant therapy after curative treatment in HCC. These results were based on a re-analysis of meta-analyses of individual patients’ data.

It was reported that surgical resection, liver transplantation, or ablation were curative treatment for HCC patients [3]. Meanwhile, an increasing number of adjuvant therapies were used to treat HCC patients. There was no HCC patient with liver transplantation included in this study because of few published study was consistent with our inclusion criteria. In addition, a lot of studies about adjuvant therapies did not provide the HR, and part of studies was not analyzed by using Kaplan-Meier survival curves and the data couldn't be extracted to calculate the HR. TACE and IFN were commonly used as adjuvant therapies after curative treatment for HCC patients nowadays. The majority of trials about TACE and IFN were conformed to the inclusion criteria. This study analyzed the published data of patients with HCC who received curative RFA combined with TACE *vs.* RFA alone, and curative treatment (surgical resection and ablation) combined with IFN *vs.* curative treatment alone.

Furthermore, cross-validation results confirmed the accurate prediction of treatment effect on OS is based on the effects on DFS for RFA + TACE *vs.* RFA and curative treatment + IFN *vs.* curative treatment. Using the STE obtained from our study to predict a non-zero treatment effect on OS, it would require the upper limit of the CI of the estimated HR to fall below 0.33, or the lower limit to be above 0.99 for RFA + TACE *vs.* RFA, and the upper limit of the CI of the estimated HR to fall below 0.87, or the lower limit to be above 1.44 for curative treatment + IFN *vs.* curative treatment. Although, both of them are wide interval for HR, the DFS as a surrogate endpoint for OS remains to be validated and it could be a better choice for the study of adjuvant therapy in HCC.

There are a few limitations to our analysis because it was based on the data extracted from literature, rather than based on the data of each patient directly. Our study included 17 trials from 8 meta-analyses, and the data extracted from Kaplan-Meier survival curves was used to calculate HRs according to a reliable method. Furthermore, with increasing clinical trials of adjuvant therapies for HCC, more work remains to be done to analyze the surrogacy of DFS for OS in upcoming study.

In conclusion, our results suggested that DFS could be used as a surrogate endpoint for OS to allow early assessment of efficacy of adjuvant therapies after curative treatment for HCC patients in future clinical trials.

## MATERIALS AND METHODS

### Search strategy and selection criteria

As presented previously [43], the search strategy was divided into two steps. First, a comprehensive automated literature search was carried out in Biosis, Embase and PubMed Databases for meta-analyses on randomized controlled trials or retrospective cohort studies that compared RFA plus TACE with RFA alone, and curative treatment plus IFN with curative treatment alone, in HCC patients. The literature search used the terms “curative treatment”, “surgical resection”, “ablation”, “RFA”, “TACE”, “interferon”, and “liver cancer” or “HCC” for studies that were published between January, 2000, and December, 2015. Eligible meta-analyses were included following the flow diagram of the PRISMA (Preferred Reporting items for Systematic Reviews and Meta-analysis) Group (2009). Second, the studies were further selected using the following inclusion criteria: (a) studies reported in English; (b) randomized controlled trials or retrospective cohort studies comparing RFA plus TACE with RFA alone, and curative treatment plus IFN with curative treatment alone for HCC; and (c) studies providing data on hazard ratios (HRs) for OS and DFS, and for those studies not providing HRs, published Kaplan-Meier survival curves of OS and DFS. Non-comparative studies that investigated IFN, RFA or TACE for HCC were excluded. The literature search and studies selection were carried out by two independent researchers. If there were any disagreements, a third researcher would decide whether the study should be included.

### Surrogacy criteria

DFS was defined as the time from randomization to the first event (loco-regional, distant recurrence, or death from any cause) after treatment. OS was defined as the time from randomization to death from any cause [14]. The surrogacy criteria were used by Marc Buyse et al. in this study [45]. The approach was based on the strength of association between the surrogate and the true endpoint (the individual-study-level surrogacy) and between the effects of treatment on the surrogate and the true endpoint (the trial-level surrogacy).

### Statistical analysis

Statistical analyses were carried out using the Matlab version R2011a and SPSS version 21.0. For each study, we extracted the data of HR (95% CI) for DFS and OS. If the study did not provide the HRs, we extracted the data from the Kaplan-Meier survival curves and calculated the HRs using the method by Jayne F Tierney et al. [46].

The association between the distribution of the true endpoint (OS) and the surrogate endpoint (DFS) was assessed by a bivariate survival model at the individual-level. To quantify the association between the effect of treatment on OS and the effect of treatment on DFS, a linear regression model was used at the trial-level. Treatment effects were estimated by hazard ratios (HRs). We classified correlation values higher than 0.9 as excellent, higher than 0.75 as very good, higher than 0.5 as good, higher than 0.25 as moderate, and equal to or lower than 0.25 as poor.

On the basis of the linear model at the second stage of the two-stage approach, we calculated the surrogate threshold effect (STE), which was defined as the minimum treatment effect on the surrogate (DFS) necessary to predict a non-zero effect on the true endpoint (OS), i.e. the HR was not equal to 1. A future trial requires an upper limit of a confidence interval for the estimated treatment effect (HR) of the surrogate to fall below the STE to predict a non-zero effect on OS, as described previously [47].

For each meta-analysis, we used a leave-one-out cross-validation approach to assess the prediction accuracy of the surrogate model. Each study was left out once and the linear model was rebuilt with the other studies. This model was then applied to the left-out study and a 95% prediction interval was calculated to compare the predicted and the observed treatment effect on OS.

## List of abbreviations

HCC: Hepatocellular carcinoma
OS: Overall survival
DFS: Disease-free survival
STE: Surrogate threshold effect
HBV: Hepatitis B virus
HCV: Hepatitis C virus
HR: Hazard ratio
OR: Odds ratio
RR: Risk ratio
RFA: Radiofrequency ablation
TACE: Transarterial chemoembolization
CT: Curative treatment
IFN: interferon
HIFU: High-intensity focused ultrasound
CI: Confidence interval
RCT: Randomized controlled trial
PS: Prospective study
RS: Retrospective study
CS: Cohort study
CCT: Case-controlled trial
PEI: Percutaneous ethanol injection
PAI: Percutaneous acetic acid
3D-CRT: Three-dimensional conformal radiation therapy
CCS: case-control study
